# Dance with spins: site-directed spin labeling coupled to electron paramagnetic resonance spectroscopy directly inside cells

**DOI:** 10.1039/d2cc05907j

**Published:** 2023-01-05

**Authors:** Annalisa Pierro, Malte Drescher

**Affiliations:** a Department of Chemistry, University of Konstanz, and Konstanz Research School Chemical Biology Universitätsstraße 10 78457 Konstanz Germany malte.drescher@uni-konstanz.de

## Abstract

Depicting how biomolecules move and interact within their physiological environment is one of the hottest topics of structural biology. This Feature Article gives an overview of the most recent advances in Site-directed Spin Labeling coupled to Electron Paramagnetic Resonance spectroscopy (SDSL–EPR) to study biomolecules in living cells. The high sensitivity, the virtual absence of background, and the versatility of spin-labeling strategies make this approach one of the most promising techniques for the study of biomolecules in physiologically relevant environments. After presenting the milestones achieved in this field, we present a summary of the future goals and ambitions of this community.

## Abstract 2

In-cell experiments require high sensitivity and resolution in complex environment. This review points out why and how SDSL–EPR spectroscopy is getting a prominent role for the study of biomolecules in living cells.

## Graphical abstract

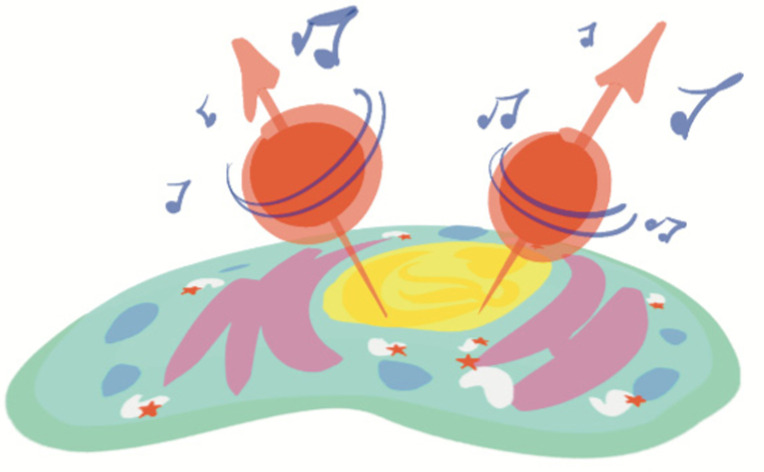

## Studying biomolecules by SDSL–EPR: from *in vitro* to in-cell studies

From the vibrational movement of biomolecules’ side chains to the condensation of the chromatin in the nuclei, biology happens on a wide timescale, from picoseconds to hours. To understand how biomolecules are interplaying during cellular processes, their structural dynamics and conformational ensembles must be characterized. Motivated by this necessity, in the last 40 years, an increasing number of techniques for studying structural dynamics at the molecular level have been developed. Among them, Electron Paramagnetic Resonance coupled with Site-Directed Spin Labeling (SDSL–EPR) progressively reached a role of relevance, extending the applications of EPR spectroscopy to diamagnetic biomolecules. Indeed, most of the cell components do not contain unpaired electrons and are EPR silent. SDSL–EPR is based on the selective introduction of paramagnetic labels, *i.e.* spin-labels, into the biomolecule of interest by site-directed mutagenesis or *de novo* synthesis, followed by its spectroscopic characterization. EPR studies can be either performed at physiological temperatures to investigate the dynamics of the labeled region, or in cryogenic conditions to characterize the structural ensemble of the biomolecule of interest.

### SDSL–EPR to probe local dynamics

**Fig. 1:**
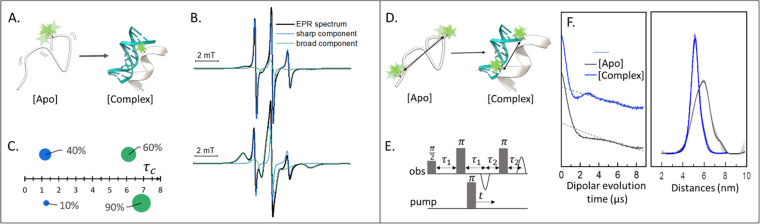
Applications of SDSL–EPR to study dynamics and conformational changes of biomolecules. (A) Scheme representing a singly-labeled protein in the absence [Apo] and presence of DNA [Complex]. (B) Simulation of typical room-temperature EPR spectra obtainable from such samples: the global simulation is reported in black, the populations with different dynamics in green (broad-feature) or blue (sharp-feature). (C) Example of a plot for the simulation results: the weight of each component is represented as the surface of the spheres while the *τ*_c_ is plotted on the *X*-axis. (D) Scheme of a doubly-labeled protein interacting with DNA ([Apo] and [Complex], respectively). (E) Four-pulses DEER sequence. (F) Exemplary raw data and distance distributions obtainable by recording the DEER traces of the [Apo] (grey) and [Complex] (blue) samples. Error barrs in the distance distributions are shown as shadows of the same color.

Singly labeled macromolecules are generally used to study the local dynamics of a labeled region (Fig. 1(A)–(C)). The spectra are sensitive to mobility of roughly five amino acid residues upstream and downstream from the labeling site into the biomolecule of interest. These experiments are performed in a continuous-wave EPR setup (also shortened as CW-EPR), in a liquid solution, at a wide range of temperatures including room temperature. By simulating the experimental data, it is possible to quantify the polarity, viscosity, and rotational dynamics of the spin-label and the spin-labeled region in terms of subpopulations (Fig. 1(C)). The first works on local spin-label dynamics appeared between 1989 and 1996.^1–5^ From that time on, this approach has been successfully used to study folding-unfolding-aggregation events,^6–8^ intermolecular regulations,^9,10^ bio-condensates and coacervates,^11–13^ changes in solvent accessibility,^14–16^ and interaction between biomolecules.^17,18^

### SDSL–EPR to study conformational ensembles

Doubly labeled biomolecules or complexes of singly labeled molecules can be used for Dipolar EPR experiments.^19,20^ One of the most powerful approaches consists in applying sequences of pulses and eventually different frequencies to detect the dipolar coupling between the paramagnetic centers and extract distribution of distances between them (*r*). The most used sequence for biological applications is named Double Electron-Electron Resonance (DEER or PELDOR),^21,22^ and consists of four-pulses at two different frequencies delayed as shown in Fig. 1(E), (for an insight on the technique see ref. 23). It is worth to mention that other pulse sequences have been developed (*e.g.* RIDME,^24^ SIFTER,^19^ using a single frequency, 5 and 7 pulse DEER…^25,26^) and can be used to improve the output of the experimental results. The dipolar coupling frequency has a *r*^−3^ dependence on the distance (*r*) between the spin-labels and is reflected in the modulation of the time traces recorded in these experiments. Consequently, simply by using chemically identical labels on both sites, it is possible to determine a distance distribution between the paramagnetic centers between 1.5 and 8.0 nm (Fig. 1(F)). In samples containing more than one biomolecule, the use of spectroscopically different spin-labels (orthogonal spin-labeling approach) allows the determination of intra-molecule and inter-molecule distances within the same sample.^27^ Differently from NMR, the size of the system under investigation does not affect the distance restraints obtainable. Indeed, the spectroscopic and relaxation properties of the system are the main limitations to the maximum distance achievable. To successfully record the modulation of the dipolar time trace, the phase memory time (*T*_m_) of the sample must be equal to or longer than the dipolar evolution period. In a standard experimental setup, this condition is achieved by performing the experiments at cryogenic temperatures (*e.g.* 10–20 K for metal centres, 40–60 K for nitroxide labels, and 80–110 K for trityl). At cryogenic temperatures, the main contribution to the *T*_m_ is the transverse relaxation of a spin-label by flip-flop transitions of neighboring protons. Therefore, by fully deuterating the solvent, cryoprotectant and the biomolecules, it is possible to expand the window of detection and extract distances up to 17 nm for proteins and 14 nm for nucleic acids.^28,29^

### Toward in-cell SDSL–EPR experiments

The design of new spin-labels has extended the applications of SDSL–EPR to the study of a wide range of macromolecules, including membrane proteins, lipids, nucleic acids and their interactions in diluted solutions. As discussed in the previous sections of this Feature Article, DEER experiments and room-temperature EPR are established techniques to study local and global dynamics, integrate structural data, and depict transient interactions between regions of a given biomolecule or between partners. Furthermore, the combination of site-directed mutagenesis, DEER experiments and multilateration approaches make it possible to use experimental data for tracking movements of whole domains of macromolecules with respect to each other.^30,31^ The upcoming challenge of the EPR community is to adapt the progress achieved in “*in vitro*” studies to “in-cell” ones. Indeed, the dynamics of biomolecules can be deeply influenced by the complexity of the cell, where transient aspecific interactions, sublocalization and environmental changes occur.

The in-cell EPR approach has been firstly demonstrated in 1956 focusing on endogenous paramagnetic centres in cells.^32,33^ Nowadays, it represents an established technique for studying Reactive Oxygen Species ROS (spin-trapping approach), tyrosyl radicals and metals complexes in cellular and animal models.^34–36^ On the other hand, protocols to exploit the advantages of paramagnetic labels for the study of diamagnetic biomolecules in cells are still under development. SDLS-EPR is particularly interesting for intracellular studies because of the negligible background signal. It opens the possibility of exploring relevant distance restraints in the nanometer range and over motional behaviour on a wide timescale (ps to ms), at physiologically relevant concentrations (nM–μM range).^37–39^

**Fig. 2:**
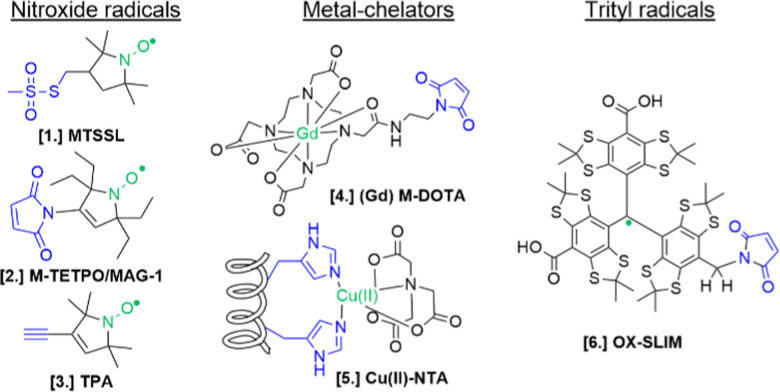
Structures of most commonly used spin-labels: nitroxides ([1] MTSSL; [2] M-TETPO/MAG-1; [3] TPA); metal-chelators ([4] M-DOTA charged with Gd(iii); [5] Cu(ii)-NTA coordinated by two Histidines); trityls ([6] OX-SLIM).^45^ The paramagnetic centres are highlighted in green, and the reactive moieties are in blue. This colour code is retained in the following figures.

For the setup of an in-cell SDSL–EPR experiment, the choice of the most appropriate spin-label is fundamental. Suitable candidates are characterized by a paramagnetic moiety that gives the spectroscopic properties of the spin-label and a grafting group specific to guarantee the specificity of labeling (highlighted respectively in green and blue in the figures of this review). The ideal spin-label should satisfy two properties: first, it should preserve the activity, folding and physiological interactions of the biomolecule on which it is grafted; second, it should not be detached or quenched by the cellular environment. Nowadays, three main families of spin labels are routinely used for *in vitro* studies (Fig. 2): nitroxyl radical derivatives (Nitroxides [1–3]), metals (Gd(iii); Cu(ii); Mn(ii) chelated by high-affinity cages [4] or protein residues like histideines [5]), triarylmethyl radicals (trityl, OX-SLIM [6]). Reviews on the advantages and limits of these spin labels can be found in the literature.^40–42^ More recently, a fourth family of photoexcitable spin-labels based on transient triplet-states induced by a laser (LaserIMD, LiDEER) has been designed.^43,44^ At the moment of writing, we are still far from seeing the latter approach exploited for cellular applications. However, being both fluorescent and paramagnetic, these spin-labels will allow the spatial localization and the structural characterization of the labeled molecule, using the same biological sample.

The design of spin-labels resistant to the cellular environment is an active field of research. In general, nitroxides, metal-chelators and trityl spin-labels are all suitable for *in vitro* and in-cell distance measurements on frozen samples. Metal-chelating cages charged with Gd(iii) are the most suitable for in-cell applications: despite the loss of signal due to the possible exchange with cellular Mn(ii) of some cages,^46,47^ they offer a narrower central field transition at higher frequencies (*e.g.* W-band) compared to nitroxides, and consequently an increase of sensitivity and reduced orientation-selection artifacts. However, the spectroscopic properties of metal-chelating labels limit the applications of in-cell SDSL–EPR to frozen solutions at which the cellular metabolism is arrested. Trityl spin-labels are the most promising candidates to open up the way for the DEER experiments at physiological temperatures, given their slow relaxation at higher temperatures. At the moment of this work, a vast majority of in-cell EPR works focus on structural studies of proteins and nucleic acids at cryogenic temperatures.

In addition to low-temperature distance measurements, nitroxide spin-labels can be used for local dynamic studies at room-temperature. The main challenge of in-cell local dynamics studies at physiological temperature is the rapid conversion of nitroxide radical into EPR-silent species (oxoammonium cations, hydroxylamines, or secondary amines) and the consequent loss of EPR signal over time.^48,49^ This problem has been partially overcome by shifting to cellular systems with a lower reduction potential (*e.g.* bacterial cells^50–52^), using oxidant agents,^53^ or substituting the methyl groups in αC-position of the radical with ethyl-groups (gem-diethyl nitroxide family; *i.e.* M-TETPO/MAG-1, Fig. 2-[2]).^54–57^ As discussed below, there are only a few examples (set to increase) in the literature using this approach to investigate biological questions.

Besides the design of new labels, an important point to address to realize in-cell SDSL–EPR experiments is how to introduce the spin-label (or the spin-labeled biomolecule) into the cell not perturbing the biological processes. The first proof-of-concept for in-cell SDSL–EPR has been published in 2010,^58^ approximately 10 years after the first in-cell NMR study.^59^ In the EPR study, the authors delivered a model protein doubly labeled with nitroxides into *Xenopus laevis* (*X. laevis*) stage IV oocytes and recorded the respective DEER trace at 60 K. Inspired by this work, the delivery of *in vitro* labeled proteins and nucleic acids into the cellular host has become the most used approach in the literature.

The results achieved using this technique are shown in the first section of this review and represent the most important insights into biological problems obtained by in-cell SDSL–EPR. In the following paragraphs, two alternative strategies to perform in-cell SDSL–EPR experiments are presented: (i) the labeling of membrane proteins located on the surface of the native cell (often called on-cell or *in situ* EPR); (ii) in-cell labeling of an overexpressed protein using non-canonical amino acids (ncAA) and orthogonal aminoacyl-tRNA synthetase (aaRS) and tRNA. Even if these strategies are still limited to the study of model proteins, they promise to be less invasive than the delivery approach on the cellular system under study. A final paragraph will, finally, list the perspectives that these works are opening, stating what we think might be the next goals of the EPR community.

## In-cell SDSL–EPR to study biomolecules: state of the art

The delivery of an *in vitro* labeled macromolecule into cells is the most used approach to address biological questions using in-cell SDSL–EPR. Indeed, performing the labeling reaction in a controlled environment (pH, salt, temperature, and label concentrations) on purified biomolecules allows high yields of labeling. Furthermore, it is also possible to deliver a de-novo synthesized molecule bearing the paramagnetic label directly in its primary sequence.

Microinjection in macroscopic cells like *X. laevis* oocytes has been the first method historically used because of the controlled and fast localization of the macromolecule in the cell cytoplasm. This approach combined with the development of labeling protocols for nucleic acids^21,22^ allowed the rapid translation of the in-cell SDSL–EPR technique from proteins to DNA^23^ and RNA.^24^

**Fig. 3:**
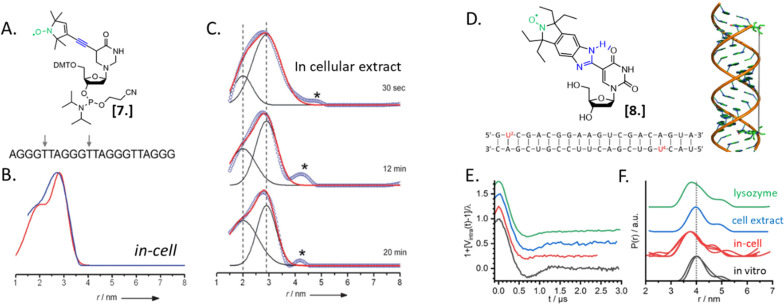
In-cell SDSL–EPR on DNA G-quadruplex and RNA duplexes. (A) Nitroxide spin-label used for the study [7] and unfolded DNA sequence injected in *X. laevis* oocytes. The labeled sites are highlighted with grey arrows. (B) Distance distributions extracted from X-band DEER of spin-labeled HT fitted with the Two-Gauss-curve model (red) and model-free Tikhonov regularization (blue); (C) time-resolved DEER distance measurement of the same DNA sequence in cellular extract: the red solid lines represent a superposition of two separate Gaussian curves (black); (D) nitroxide spin-label ElmUm [8], RNA sequence and structure used in the study; the modified Uracyl base is highlighted in the sequence in red; (E) background-corrected DEER data normalized by the modulation depth; (F) distance probability distributions obtained by model-free analysis for the duplex RNA (multiple traces show results obtained from different samples). Adapted with permission from M. Azarkh, V. Singh, O. Okle, D. R. Dietrich, J. S. Hartig and M. Drescher, *ChemPhysChem*, 2012, **13**, 1444–1447, © 2012 Wiley-VCH Verlag GmbH & Co. KGaA, Weinheim; and from A. Collauto, S. Bülow, D. B. Gophane, S. Saha, L. S. Stelzl, G. Hummer, S. T. Sigurdsson and T. F. Prisner, Angew. Chem*.,* Int. Ed., 2020, **132**, 23225–23229. ©2020 Published by Wiley-VCH GmbH.

**Fig. 4:**
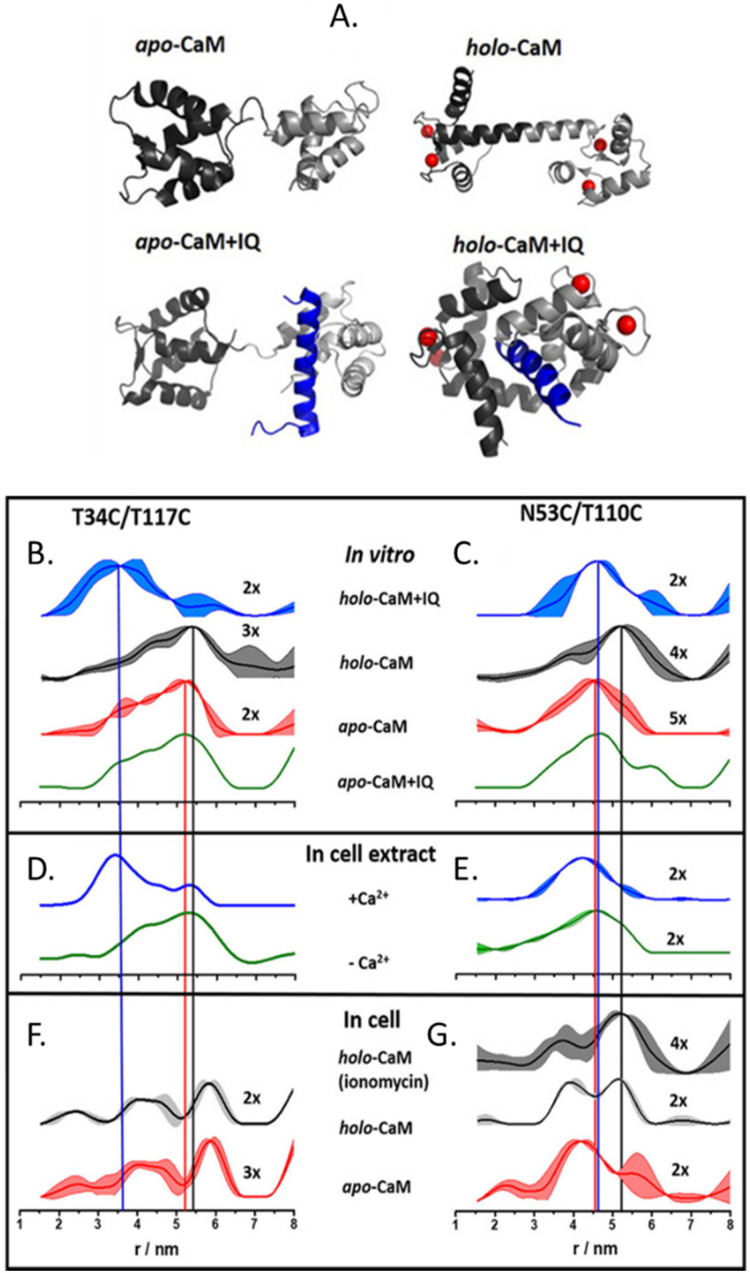
Conformational changes of CaM *in vitro*, in cellular extract, and in cells. (A) Possible conformations of CaM: Ca(ii) ions are represented in red, and the peptide partner “IQ” in blue. Distances distributions extracted from *in vitro* experiments are in panels (B) and (C); in HeLa cellular extracts (D), (E) and in HeLa cells in panels (F) and (G) The data corresponding to the apo-protein is shown in red, in the presence of Ca(ii) ions (holo-CaM) in grey, of the IQ peptide in green, and of both in blue. Adapted from A. Dalaloyan, A. Martorana, Y. Barak, D. Gataulin, E. Reuveny, A. Howe, M. Elbaum, S. Albeck, T. Unger, V. Frydman, E. H. Abdelkader, G. Otting and D. Goldfarb, *ChemPhysChem*, 2019, 20, 1860–1868, ©2019 Wiley-VCH Verlag GmbH & Co. KGaA, Weinheim.

**Fig. 5:**
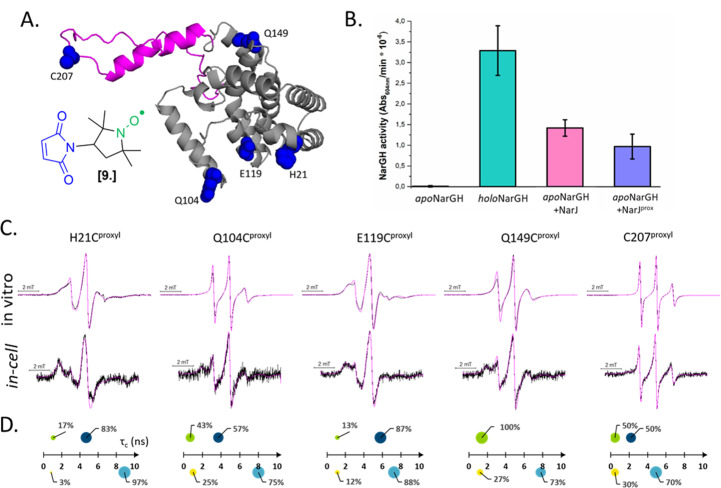
In-cell EPR study of a protein in its native host. (A) Structure of the nitroxide M-proxyl [9] and AlphaFold predicted structure of NarJ: the positions targeted for cysteine mutations are highlighted in blue, the c-terminal disordered tail in pink. (B) In-cell activity test of NarJ delivered in *E. coli* cells expressing the protein partner: inactive apoNarGH prior to delivery of NarJ (negative control, black); holoNarGH (positive control, green); apoNarGH activated by unlabeled NarJ (pink) or labeled NarJ^prox^ (purple). (C) Room temperature, X-band CW-EPR spectra recorded for the NarJ *in vitro* and in *E. coli* cells. Experimental data are in black, simulated in magenta. (D) results of the spectral simulations: *in vitro* data are above the axis (green and navy), in cell are below (yellow and cyan). The two components extracted from each spectrum are represented as spheres whose surface reports their percentage, while their position on the *X*-axis the *τ*_c_ (ns). Adapted with permission from Pierro, A., Bonucci, A., Normanno, D., Ansaldi, M., Pilet, E., Ouari, O., Guigliarelli, B., Etienne, E., Gerbaud, G., Magalon, A., Belle, V. and Mileo, E. (2022), Probing Structural Dynamics of a Bacterial Chaperone in Its Native Environment by Nitroxide-Based EPR Spectroscopy. *Chem. Eur. J.* 2022, **28**, e202202249, © 2022 The Authors. Chemistry – A European Journal published by Wiley-VCH GmbH.

**Fig. 6:**
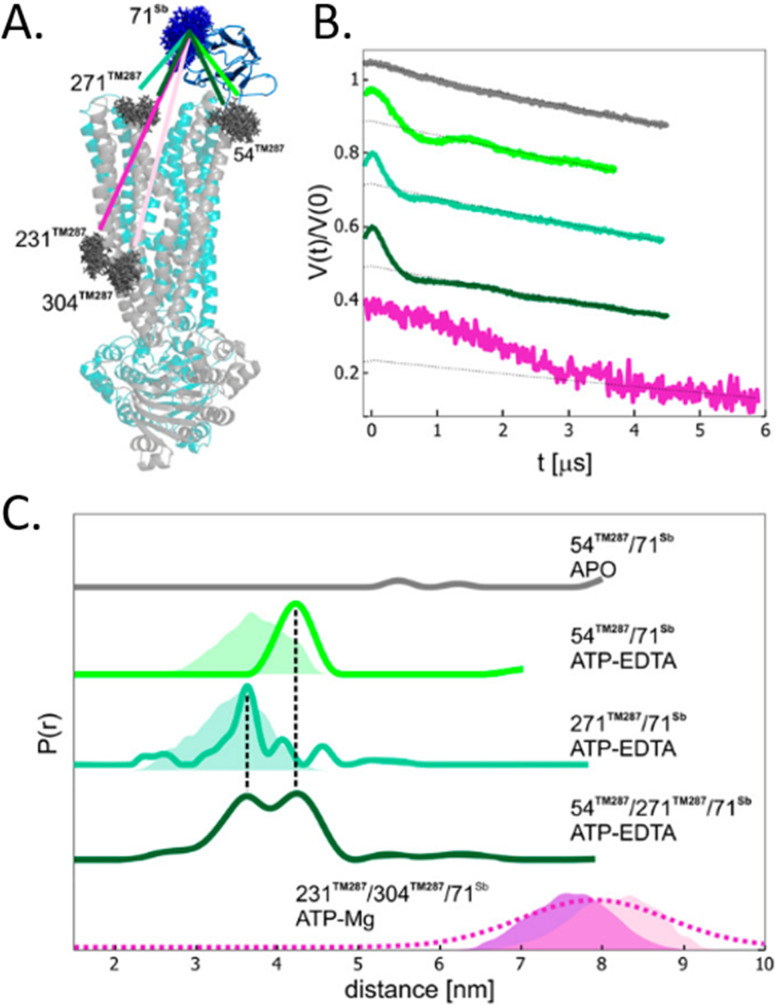
Sybody (Sb) labeling of the ABC transporter *via* Gd–nitroxide distances. (A) Crystal of the protein labelled with MTSSL (gray) in the presence of the Sybody labeled with (Gd)M-DOTA (blue). (B) DEER traces of protein labeled with Sybody-Gd and MTSSL. (C) Distance distributions of three variants of the transporter in the apo-form and in the presence of ATP-EDTA and ATP-Mg. Simulated distance distributions are shown as shaded areas, the one for 231TM287-71Sb is displayed in purple, 304TM287-71Sb in pink. The distribution obtained by Gaussian model fit is shown in dotted lines. Adapted with permission from L. Galazzo, G. Meier, M. Hadi Timachi, C. A. J. Hutter, M. A. Seeger and E. Bordignon, *Proc. Natl. Acad. Sci. U. S. A.*, 2020, **117**, 2441–2448 ©2020 National Academy of Sciences.

**Fig. 7:**
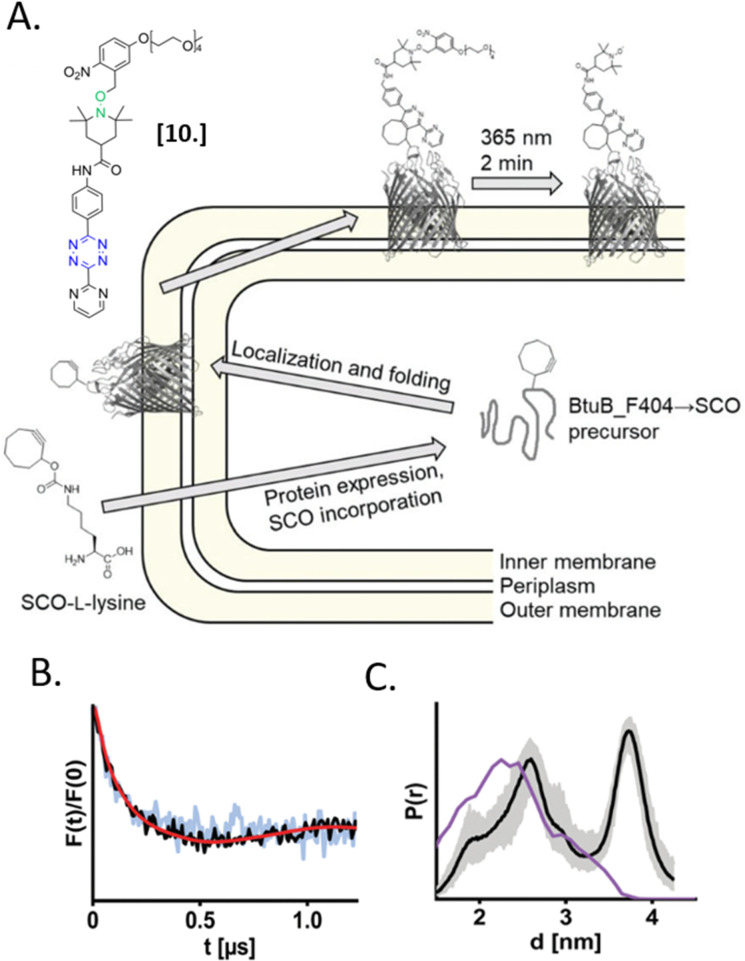
*In situ* EPR on BtuB protein combining ncAA genetic-code expansion and photo-induced nitroxide PaNDA [10]. (A) Schematic representation of the protein expression and spin-labeling reaction; (B) background corrected DEER traces of BtuB-SCO-PaNDA in the presence of the TEMPO-CNCbl in *E. coli* cell membrane (black) or in isolated outer membranes (light blue). (C) Corresponding *in situ* distance distribution (black, error bar in grey). The purple line indicates the simulated distance distribution. Reproduced from A. Kugele, S. Ketter, B. Silkenath, V. Wittmann, B. Joseph and M. Drescher, *Chem. Commun.*, 2021, **57**, 12980–12983 with permission from the Royal Society of Chemistry.

Nucleic acids’ secondary structures play a crucial role in their regulation and translation; therefore, they are among the most appealing target for in-cell studies. One of the first biological problems addressed using in-cell SDSL–EPR is the possible conformations of Human Telomeric (HT) G-quadruplexes in the cellular environment (Fig. 3).^25^ Before this study different structures had been resolved in the presence of Na^+^ or K^+^ salts, but it was not clear which one was physiologically relevant. The authors of this study microinjected a salt-free solution of unfolded (but labeled, Fig. 3(A)-[7]) HT-DNA sequence into *X. laevis* oocytes. After 15 minutes of incubation at room temperature, they recorded X-band DEER traces of the oocytes in frozen solution. By fitting these data, it was possible to estimate the coexistence of a 1 : 1 parallel propeller quadruplex and an antiparallel basket quadruplex (Fig. 3(B)). The progressive folding into these two conformations has also been studied over different time windows by recording DEER traces of the same DNA sequence incubated in cellular lysates (Fig. 3(C)). Comparing these data with previous studies, the authors found that the physiological environment has an impact similar to what was obtained *in vitro* in the presence of K^+^ ions.

**Fig. 8:**
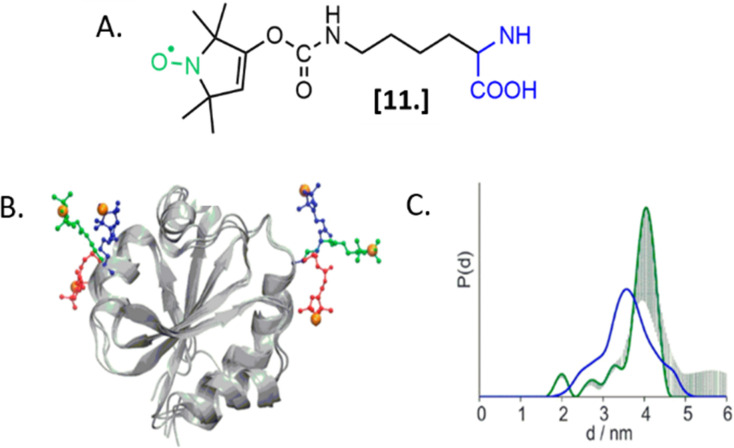
Genetic encoding of spin-labeled amino acid SLK-1 and intramolecular EPR distance measurements in *E. coli* on TRX protein. (A) SLK-1 spin-label [11]; (B) structure of the Thioredoxin (pdb: 2TRX) highlighting the rotamers of SLK-1 as red, green and blue sticks; (C) distance distribution for Thioredoxin labeled on the residues D14/G34SLK-1 (green, error bars in grey) compared to the corresponding theoretical distance distribution predicted on the basis of the rotamer library (blue). Adapted with permission from M. J. Schmidt, J. Borbas, M. Drescher and D. Summerer, *J. Am. Chem. Soc.*, 2014. © 2014 American Chemical Society.

In 2020, Collauto *et al.* designed a rigid *gem*-diethyl nitroxide specific for the uracil base of RNA (Fig. 3(D)-[8]). Q-band DEER experiments on an RNA duplex in the cytoplasm of *X. laevis* oocytes revealed a reproducible decrease of the main distance between the labeled sites on the RNA duplex suggesting a compacting of the structure (Fig. 3(E) and (F), black and red curves). Noteworthy, this change was not found in extracts but seemed triggered by positively charged lysozyme solution used as a crowding agent (Fig. 3(E) and (F), blue and green curves).

Despite the strong contributions microinjection gave to this research field, the EPR community has been progressively shifting toward bulk-delivery techniques which can be extended to other cellular systems than *X. laevis*. Labeled proteins have been successfully internalized *via* cell-phagocytosis;^60^ penetration properties of the biomolecule under investigation;^61^ or induced transient permeability of the membrane using osmotic pressure,^46,62,63^ thermic shock^50^ or transient electric fields.^52,63,64^ These methods have been successfully applied to record DEER traces in eukaryotic cells to study conformational ensembles,^62,65,66^ destabilization of a homodimer,^47^ or the effect of a ligand.^67^

The more in-cell EPR studies have been published, the more evidence emerged that the impact of the cellular environment cannot be simulated *in vitro*, neither by crowding agents (also known as *crowders*) nor by cellular lysates. One work showing the limits of the *in vitro* approach is the comparison of the structural conformations of the human calmodulin (CaM) labeled with (Gd)M-DOTA (Fig. 2-[4]) on cysteine residues in eukaryotic HeLa cells, *in vitro*, in crowders, and cellular lysates.^67^ As shown in Fig. 4, there are four known conformational states of CaM triggered by the binding with Ca(ii), by a partner peptide IQ, or by the presence of both. While the DEER traces obtained *in vitro* (Fig. 4(B) and (C)) and in cell extract (Fig. 4(D) and (E)) showed a similar distance distribution in the presence and in the absence of Ca(ii), the in-cell one was broader showing higher flexibility of the protein when in a physiological context.

CaM in the cell extract seems to interact aspecifically with a partner impacting the conformational ensemble of the protein. The lack of this interaction (or of these conformational changes) inside the cells (Fig. 4(F) and (G)), demonstrates that in-extract data can give misleading information if not complemented with ones obtained in intact cells.

A further step towards “physiological” in-cell SDSL–EPR consisted of studying room-temperature local dynamics in a cellular context. This approach exploits the ability of nitroxides in reporting backbone dynamics at a wide range of temperatures, including physiological ones. In 2017, Cattani *et al.* realized the first proof-of-concept of this approach by microinjecting in *X. laevis* oocytes the amyloidogenic protein α-synuclein.^68^ For several years no studies followed this path, limited by the short persistence of these radicals in-cell and the long dead-time between the delivery-trigger and the first EPR spectrum for mammalian cells (*i.e.* 45 minutes–18 hours).^64^ The development of fast and efficient delivery protocols for bacterial cells (10–15 minutes) and the longer half-life of nitroxides in this environment allowed room temperature CW-EPR using nitroxides to study biological problems.^50–52,56^

Combining *in vitro* labeling and electroporation delivery, Pierro *et al.* published in 2022 the first room-temperature characterization of a cytoplasmic protein in its native environment (NarJ in *Escherichia coli* (*E. coli*) cells, Fig. 5(A)).^52^ After demonstrating that the delivered protein can activate its partner into the cell (Fig. 5(B)), the authors investigated the local and global dynamics of the protein by EPR. By labeling different regions of the protein with M-proxyl nitroxide [9] and simulating the CW-EPR spectra obtained *in vitro* and in-cell, it was possible to quantify the impact of the cellular environment on the local dynamics of the protein in terms of rotational spin label dynamics. The authors pointed out that the cell has a site-specific impact on protein mobility, and similar results have been reproduced *in vitro* in the presence of crowding agents. Being performed at room temperature, those measurements open the way for time-resolved EPR, as discussed in the perspective section.

Even if the delivery has undeniable advantages, this approach of *in vitro* labeled biomolecules has several limitations. Firstly, it is impossible to exclude the impact of the delivery trigger itself on the cell physiology and, consequently, on the global interactome of the biomolecule under investigation. Secondly, the current bulk method does not allow a defined and uniform localization of the protein in the cell population under investigation (*e.g.* concentration, organelle sublocalization, equal efficiency of transfection of cells at different growth stages). For these reasons, complementary and alternative methods are under development.

## On-cell EPR

On-cell EPR (or *in situ* EPR) is an interesting alternative to the delivery approach to investigate surface-exposed membrane proteins directly in their native lipid environment. The target protein, bearing one or multiple surface-exposed amino acids (generally cysteines), is overexpressed in *E. coli* and the spin-labeling reaction is performed directly on intact cells. After removing the excess of spin-label, the cell suspension is, then, frozen and the respective EPR experiments are carried out. The specificity of labeling is based on the assumption that membrane proteins are naturally poor in cysteines, and that the spin-labels will be quenched in the cytoplasm.^69^ Consequently, nitroxides are the most used spin labels for this application, even if a recent study demonstrated that also trityls can be successfully used.^51^

Using on-cell EPR approach, it was possible to study the structural change of the cobalamin membrane transporter BtuB of *E. coli* in its native cellular environment.^69,70^ Recording DEER traces in the presence of BtuB doubly labeled with the nitroxide MTSSL ([1], Fig. 2) and its ligands (Ca(ii) ions and Cyanocobalamin), conformational changes in coherence with the reported crystals were detected. Unfortunately, on-cell EPR studies are limited to proteins with high expression yields. Indeed, low overexpression yields often affect the specificity of the labeling and the consequent background signal from the native membrane proteins.

To avoid aspecific cysteine labeling, in 2020 Galazzo *et al.*^71^ tested in cellular lysates and *in vitro* a strategy relying on the use of nanobodies (∼15 kDa, also called Sybody) carrying on a cysteine residue a (Gd)M-DOTA (Fig. 2-[4]) spin-label (Fig. 6(A)). As full-length antibodies, nanobodies are characterized by a high specificity for protein aptamers, guaranteeing a high selectivity of labeling even in complex environments. To prove the adaptability of the Sybody as a spin-probe, the study has been mainly carried out in solution using native ABC transporter proteins in detergent, measuring DEER traces between (Gd)M-DOTA on the Sybody and MTSSL (Fig. 2-[1]) on the protein cysteines (Fig. 6(B) and (C)). Once proved that was possibleto extract differences in distance distributions in the presence or absence of the ligands of the transporter (ATP, Mg(ii)) *in vitro*, the authors tested the Sybody labeling on membranes extracted directly from *E. coli* cells overexpressing the protein. The main limitation of this approach is that the protein under investigation must be significantly bigger than the nanobody itself. Furthermore, the physical distance between the spin-label and the “labeled” protein can affect the sensitivity of this approach to relatively small conformational switches. Nevertheless, the use of Sybody is particularly promising for the study of membrane-embedded proteins in hosts other than *E. coli*, where high overexpression yields are challenging to achieve.

In 2021, Kugele *et al.* proposed an alternative approach to gain specificity of labeling in on-cell EPR experiments. In this work, the authors achieve the bio-orthogonality of the reaction by introducing a ncAA (SCO-l-lysine, Fig. 7(A)) on the surface of the model protein BtuB. More details about a genomic expansion using ncAA and spin-labels reactivity will be discussed in the following paragraph. Using the photoactivable nitroxide named “PaNDA” specific for the SCO-l-lysine (Fig. 7-[10]),^72^ it was possible to measure a DEER trace between the PaNDA and a paramagnetic analog of the native ligand, cobalamin (TEMPO-CNCbl). Besides the interest of using ncAA on the cell surface, the possibility of activating the nitroxide radicals by irradiation at 365 nm for few minutes, extends the time window of nitroxide resistance even further and opens up the way for more studies at room temperature in the membrane environment.

## In-cell spin-labeling

The labeling of biomolecules directly in their physiological environment is the highest dream of in-cell SDSL–EPR community. This would allow the study of biomolecules without any purification step, with the minimum stress for the cell, virtually preserving the folding, the post-translational modifications, and the activity of the biomolecule of interest. As for *in vitro* studies, the choice of the spin-label is crucial. In this experimental setup, the spin-label must penetrate the membranes and selectively graft the residue(s) of interest in this complex environment, at that defined pH and redox potential. Consequently, extensive research is currently going on to achieve high yields of specific labeling using cell-resistant spin-labels, simultaneously avoiding cytotoxicity.

The first ambitious and revolutionary work in this field was published in 2014.^73^ Starting from a lysine analog, Schmidt *et al.* synthesized a ncAA bearing a paramagnetic nitroxide function (SLK-1 [11], Fig. 8(A)) able to be encoded directly in the protein of interest using appositely evolved tRNA^Pyl^ and pyrrolysyl-tRNA-synthetase pairs. Once optimized, this metal catalyst-free one-step labeling was used to record the first distance distribution of an in-cell labeled (and afterwards purified) protein Thioredoxin (Fig. 8(B) and (C)).

Because of the challenging evolution of the tRNA^Pyl^/polymerase pairs for paramagnetic amino acids and the short persistence of SLK-1 in the cellular environment, this approach has not yet been used in more complex protein systems. As often happens in science, this example opened the way for a significant number of papers aiming at developing a two-step in-cell labeling. Here, the selectivity of grafting is guaranteed by encoding a diamagnetic ncAA in the protein of interest *via* an amber stop codon (TAG). The latter is selective for a customized spin-label incubated in the cell suspension after the overexpression of the protein. Once in contact with the intact cells, the spin-labels penetrate the membranes and, in the presence of a bio-compatible catalyst (*e.g.* copper, palladium), reacts with the ncAA.^74,75^ More recently a catalyst-free reaction based on Diels–Alder chemistry has been developed to avoid possible cytotoxicity of the metals used for previous reactions.^72,76^ For an insight on the spin-label ncAA pairs available, we recommend a recent review at the ref. 77.

One of the most used reactions for in-cell labeling is based on azide-functionalized spin-labels incubated with cells overexpressing proteins bearing azide- or alkyne-ncAA. These couples are reacting directly inside the cell *via* copper(i)-catalyzed azide-alkyne cycloadditions (CuAAC). To our knowledge, the first in-cell study performed with this technique used azide-functionalized nitroxide and the ncAA *N*-ε-propargyl-l-lysine (PrK). After purification of the in-cell labeled protein, a second nitroxide was introduced *in vitro* and a DEER trace was recorded.^78^ Nowadays, a wide range of nitroxides and Gd(iii)-based spin labels have been synthesized, and the labeling yield of CuAAC reaction has been increased up to 85%.^56,76^

**Fig. 9:**
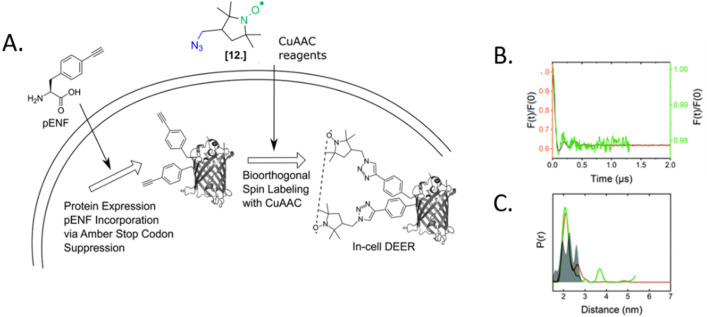
In-cell DEER on an *in vivo* labeled protein. (A) Schematic overview of the *in vivo* spin labeling approach *via* incorporation of ncAA pENF in eGFP, copper-catalyzed labeling followed by in-cell EPR distance determination. (B) Comparison of the form factors of Y39/L221pENF-L eGFP from DEER measurements conducted *in vitro* (orange) or *in vivo* (green). (C) Derived distance distribution for DEER measurements *in vitro* (orange) and *in vivo* (green). The gray area indicates the expected distance distribution based on MMM calculations. Reproduced from P. Widder, J. Schuck, D. Summerer and M. Drescher, *Phys. Chem. Chem. Phys*, 2020, **22**, 4875, with permission from the Royal Society of Chemistry.

The only in-cell labeling and in-cell DEER published at the moment was performed by Widder *et al.* in 2020.^75^ The overexpressed eGFP containing two *para*-ethynyl-l-phenylalanine (pENF, Fig. 9) ncAA was labeled in *E. coli* cells incubated with CuAAC and the specific nitroxide. The DEER traces led to distances comparable to those obtained *in vitro* for this model protein, as expected for an intact biomolecule. This work is the first proof that the two-step labeling using ncAA technology can be used for performing experiments directly in intact cells.

Generally speaking, this approach is still restricted to methodological studies: the complex setup of the labeling protocol, the low yields of labeling for proteins other than model ones and the challenging overexpression of full-length proteins containing multiple stop codons are among the causes. However, the promising studies mentioned and the progress achieved in biorthogonal labeling suggest that this branch is in further development.

## Quo vadis, in-cell EPR?

The EPR community in the last decade successfully demonstrated the applicability of SDSL–EPR to in-cell experiments. From the dynamics of nucleic acids to the transient assembly of proteins, this approach is suitable to study biological processes virtually without background interference and size restriction of the biomolecule under investigation. This latter point is particularly interesting if applied to the study of in-cell interactions between biomolecules, where NMR is severely affected by the loss of signal. Consequently, one of the most ambitious perspectives for in-cell SDSL–EPR is gaining prominence in studying large systems and their interactions at the local and global levels. We still need to walk a long way to get there. The technique is progressively evolving towards less invasive approaches to introduce the spin-labels inside the cell and more physiologically relevant experiments. It has been demonstrated that in-cell experiments at 200 nanomolar are possible using Gd-chelators;^37^ and even lower concentrations have been achieved using copper tags *in vitro*.^38^ This will lead to experimental conditions able to preserve the native stoichiometry of aspecific and specific interactions inside the cell. In parallel with this research path, the community is working on the study of Post-Translational Modifications (PTMs, *i.e.* labeling of native O-GlcNAcylation sites) in a living cell, for which a limited number of methods are available. The EPR quantification of PTMs and the use of these sites for structural studies are among the most exciting perspectives of the next years.^79,80^

The optimization of in-cell labeling strategies in bacteria and eukaryotic cells, and the use of native promoters will allow to study biomolecules in their native hosts, at the correct localization, possibly in cells synchronized at the same metabolic stage in the next future. At the moment, the only spin-labels able to permeate bacterial membranes for in-cell labeling are nitroxides. Nitroxides’ undeniable advantage is the possibility of using the same label for both room-temperature local dynamics study and cryogenic distance determinations. However, the limited (but improved) persistence of the paramagnetic function has to be taken into account when designing the experiment. The design of permeable metal-based and trityl spin-labels will expand the time window of pulse EPR experiments: it will be possible to label a biomolecule directly into bioreactors analogously to what is done in in-cell NMR experiments.^81^

The transition towards more biocompatible conditions passes through the study of biological processes at physiological temperatures. Extensive work is currently done to increase the accessible temperature range for pulse EPR experiments *in vitro*.^82–85^ At the moment, good results have been achieved using trityl spin-labels, demonstrating the potential applications of these carbon-centered radicals in the cellular context.

Recently the community also focused on the development of in-cell time-resolved experiments to study the changes in protein dynamics in living organisms. In one example, the reduction rate of nitroxides has been used to perform reduction kinetics in bacterial cells. This approach allowed tracking dynamics and accessibility to reducing agents with a time resolution of maximum 10 minutes.^52,86^ The development of a commercially available Rapid-scan setup coupled with in-cell EPR will improve the time resolution up to ms μs^−1^ according to the *T*_2_ of the sample, increasing up to 17 times the signal-to-noise ratio.^87^ The first proof-of-concept in this matter has been recently published, demonstrating that is possible to follow the interaction of alpha-synuclein and lipids in *X. Laevis* oocytes.^88^ However, the complex data elaboration and the problematic background correction still make the rapid-scan experiments particularly challenging.

All the milestones achieved by in-cell SDSL–EPR must be, finally, considered in the context of a common “integrative” structural biology project. Understanding a complex event such as the dynamics of a biomolecule in the presence of its interactome in the cellular environment needs a multidisciplinary approach. The combination of different techniques is, therefore, fundamental. The data obtained from different techniques can be ultimately integrated *in silico* and finally describe the behavior of a biomolecule in a living cell. At the moment of this work, in-cell integrative biology experiments are still challenging but already possible on model systems. Keeping in mind the *vision* of integrative structural biology, we will be able to adapt the biophysical techniques used *in vitro* (improve their biocompatibility) and study biology in native conditions.

## Author contributions

The authors equally contributed to the conceptualization of this work. A. P. wrote the original draft, M. D. reviewed and edited the work.

## Conflicts of interest

There are no conflicts to declare.

## Supplementary Material
